# Dysregulation of PAK1 Is Associated with DNA Damage and Is of Prognostic Importance in Primary Esophageal Small Cell Carcinoma

**DOI:** 10.3390/ijms160612035

**Published:** 2015-05-27

**Authors:** Jinfeng Gan, Yuling Zhang, Xiurong Ke, Chong Tan, Hongzheng Ren, Hongmei Dong, Jiali Jiang, Shaobin Chen, Yixuan Zhuang, Hao Zhang

**Affiliations:** 1 Cancer Research Centre, Shantou University Medical College, Shantou 515063, China; E-Mails: gjf88900406@126.com (J.G.); kxr_2014_88900406@126.com (X.K.); dhm88900406@126.com (H.D.); jjl_2014_88900406@126.com (J.J.); 2 Department of Information, Affiliated Cancer Hospital of Shantou University Medical College, Shantou 515031, China; E-Mail: zyl88900406@126.com; 3 Department of Biotherapy, Affiliated Cancer Hospital of Shantou University Medical College, Shantou 515031, China; 4 Department of General Surgery, the Fifth Affiliated Hospital of Sun Yat-sen University, Zhuhai 519000, China; E-Mail: cht_22003@126.com; 5 Department of Pathology, Central Hospital of Kaifeng, Kaifeng 475000, China; E-Mail: hongzh_ren@126.com; 6 Thoracic Surgery, Affiliated Cancer Hospital of Shantou University Medical College, Shantou 515031, China; E-Mail: shbin2442_c@126.com; 7 Tumor Tissue Bank, Affiliated Cancer Hospital of Shantou University Medical College, Shantou 515031, China; E-Mail: zyx88900406@126.com

**Keywords:** primary esophageal small cell carcinoma, p21-activated kinase 1, DNA damage, γH2AX, metastasis, prognosis

## Abstract

Primary esophageal small cell carcinoma (PESCC) is a rare, but fatal subtype of esophageal carcinoma. No effective therapeutic regimen for it. P21-activated kinase 1 (PAK1) is known to function as an integrator and an indispensable node of major growth factor signaling and the molecular therapy targeting PAK1 has been clinical in pipeline. We thus set to examine the expression and clinical impact of PAK1 in PESCC. The expression of PAK1 was detected in a semi-quantitative manner by performing immunohistochemistry. PAK1 was overexpressed in 22 of 34 PESCC tumors, but in only 2 of 18 adjacent non-cancerous tissues. Overexpression of PAK1 was significantly associated with tumor location (*p* = 0.011), lymph node metastasis (*p* = 0.026) and patient survival (*p* = 0.032). We also investigated the association of PAK1 with DNA damage, a driven cause for malignancy progression. γH2AX, a DNA damage marker, was detectable in 18 of 24 (75.0%) cases, and PAK1 expression was associated with γH2AX (*p* = 0.027). Together, PAK1 is important in metastasis and progression of PESCC. The contribution of PAK1 to clinical outcomes may be involved in its regulating DNA damage pathway. Further studies are worth determining the potentials of PAK1 as prognostic indicator and therapeutic target for PESCC.

## 1. Introduction

Primary esophageal small cell carcinoma (PESCC) is a “rare type” cancer or a rare sub-type of esophageal cancer [1]. PESCC is characterized by poor prognosis and metastasis is dominated at the time of initial diagnosis, which is aggressive esophageal carcinoma associated with a fatal clinical outcome [1,2]. Compared with the more common counterpart, PESCC has been traditionally understudied. Our limit knowledge is often derived from case reports, usually from single institute. Many molecular events that drive the tumor spreading and metastasis are barely explored in PESCC. This has largely led to a lack of evidence upon which to base targeted treatment.

The DNA double-strand breaks (DSBs) are serious lesions that can cause genomic instability, ultimately leading to cancer [3]. Increased levels of DSBs have been demonstrated in tumor cells in cancer specimens, as well as in tumor cells *in vitro* [4,5,6,7], and leading to cancer cell invasion, which is important for metastasis [8]. γH2AX, a key regulator of DNA damage response (DDR), has been recently demonstrated to be significantly correlated with lymphatic infiltration in non-small cell lung cancer [9]. In addition, γH2AX was a poor prognostic indicator in non-small cell lung cancer [9], endometrial carcinomas [10] and triple-negative breast cancer [11]. More recently, another study has demonstrated that γH2AX was significantly enriched in metastatic renal cell carcinoma, and measurements of γH2AX was a potentially useful mean in clinical diagnosis of metastatic renal cell carcinoma [12].

p21-activated kinase 1 (PAK1), a ubiquitous serine/threonine protein kinase that is widely upregulated in human cancers and plays fundamental role in cellular processes such as invadopodia disassembly [13], cell adhesion and migration [14] and processes that are relevant to metastasis and progression [15,16], epithelial to mesenchymal transition (EMT) [17]. PAK1 acts as a convergence point in a composite oncogenic signaling pathway [18] and its dysregulation possibly influences oncogenic transformation and survival. Several clinical [19,20] and functional studies have implicated PAK1 in tumor metastasis. Studies confirmed that inhibition of PAK1 in human tumors and *in vivo* tumor models resulted in an anti-tumoral effect [15]. The clinical pipeline of molecular therapy targeting PAK1 using small molecular inhibitors has been growing [21,22,23,24,25,26]. Recently, the role of the PAK1 in DDR has begun to emerge, PAK1 has recently been demonstrated to phosphorylate microrchidia CW-type zinc finger 2 (MORC2), resulting in induction of γH2AX [27] and plays an important role in gene expression modulations that were associated with DNA damage induced by ionizing radiation [28].

Of note, PAK1 is upregulated in cancers of gastrointestinal tracts including colorectal, gastric cancer and gastroesophageal junction adenocarcinoma [19,29,30]. In particular, PAK1 overexpression has been found in small cell lung cancer (SCLC), a far more common type of small cell carcinoma [15]. However, clinical importance of PAK1 in PESCC and the correlation between PAK1 and DDR in surgically resected specimens remains unknown. Hence, this encourages us to study the clinical importance of PAK1, and investigate the relationship between the expression of PAK1 and γH2AX in PESCC. We evaluated PAK1 and γH2AX expression using immunohistochemistry in PESCC, corresponding adjacent non-cancerous tissues and correlated PAK1 with clinical variables, and γH2AX. We observed that PAK1 overexpression was significantly associated with tumor location, lymph node metastasis and reduced overall survival, and PAK1 expression is also associated with γH2AX. Collectively, this data opens new avenues of understanding the clinical relevance of PAK1 in progression and metastasis of PESCC, and a potential role of PAK1 in genomic instability.

## 2. Results and Discussion

### 2.1. Patient Characteristics

The median age of the patients was 58.50 and it ranges from 42 to 79. Study consisted of 24 males and 10 females with a male to female ratio of 12:5. Patients underwent transthoracic esophagectomy with two-field lymphadenectomy. None of the patients underwent chemotherapy or radiotherapy before surgical resection of tumor and none had prior malignant or distant metastasized tumor on routine examination before surgery.

### 2.2. Tumor Characteristics

Tumor length varied from 0.6 to 12 cm, with an average length of 5 cm. No information is available for 2 patients on tumor length. A total of 22 tumors were located in the middle third of the esophagus, 7 were on the lower third of the esophagus and 5 were on the upper third of the esophagus.

### 2.3. Staging and Treatment

Of the 34 patients, 3 patients belonged to stage Ib, 2 patients belonged to stage IIa, 15 patients belonged to stage IIb, 5 patients belonged to stage IIIa, 2 patients belonged to stage IIIb and 3 patients belonged to stage IIIc. No information is available on stage of cancer for 4 patients. Surgery, chemotherapy and radiotherapy were given as treatment. Etoposide combined with cisplatin was the most common treatment regimen in chemotherapy. A total of 34 patients received treatment, of them 18 patients received curative surgery, 9 patients received surgery followed by chemotherapy, 2 patients received surgery followed by radiotherapy, 3 patients received surgery followed by chemo-radiotherapy and 2 patients received chemotherapy only.

### 2.4. PAK1 Is Up-Regulated in Primary PESCC vs. Adjacent Non-Cancerous Tissues

**Figure 1:**
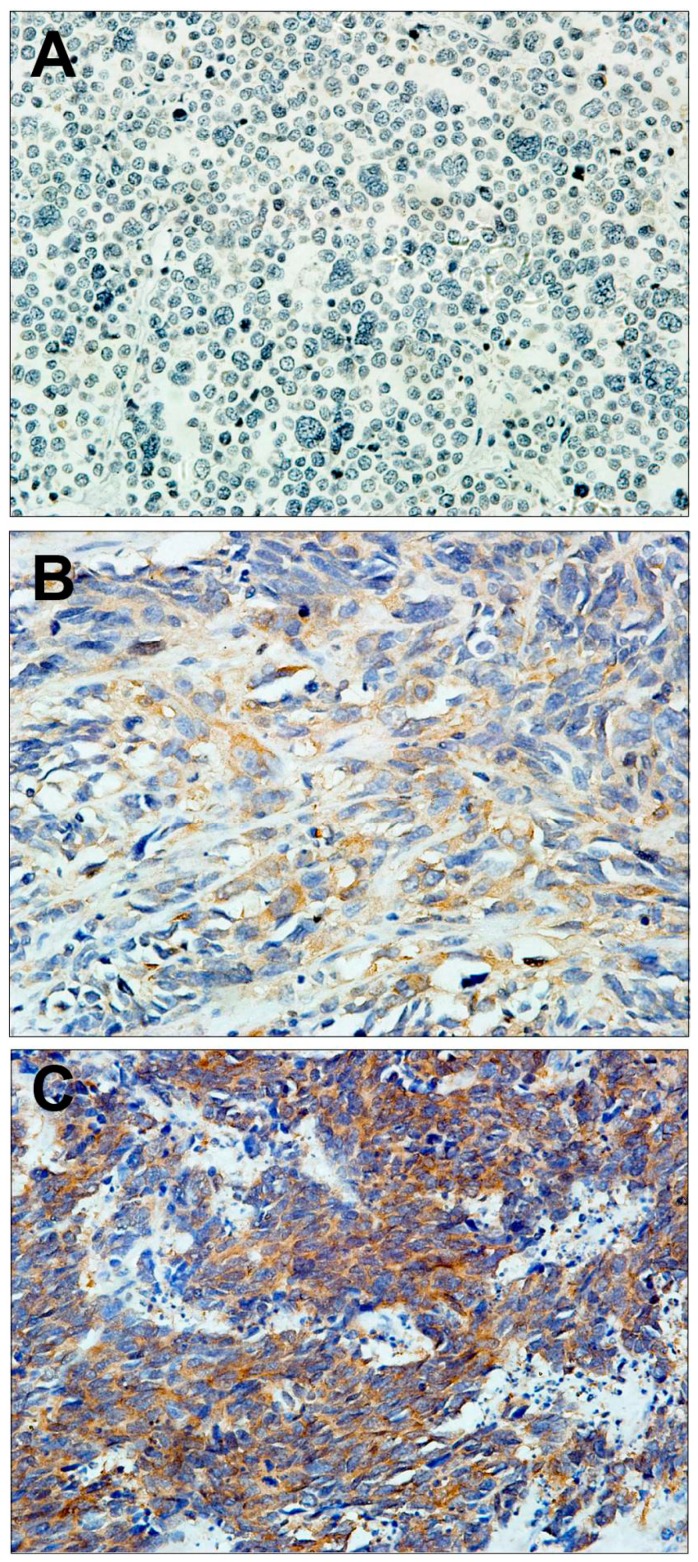
Representative serial sections of PESCC (Primary esophageal small cell carcinoma) immunohistochemiscal staining for PAK1: (**A**) negative staining; (**B**) weak staining; and (**C**) strong staining. Magnification: 400×.

PAK1 protein, which is shown as brown granules in Figure 1B, Figure 1C, was mainly localized in the cytoplasm. Of the 34 PESCC samples analyzed, 22 (64.71%) were grouped as positive and 12 (35.29%) were grouped as negative (Figure 1A), based on the composite histoscore. Of the 18 adjacent non-cancerous tissues analyzed, 2 (11.11%) were grouped as positive and 16 (88.89%) were grouped as negative. Comparison of expression level of PAK1 between tumor and adjacent normal tissue showed a statistically significant increase in tumor tissues (*p* = 0.000, Table 1).

**Table 1:** Correlation between PAK1 overexpression and clinicopathological variables in primary esophageal small cell carcinoma.

Clinical Variables	Total Number	PAK1 Overexpression		
		Negative	Positive	*p* Value
Gender				
Male	24	7	17	0.271
Female	10	5	5	
Age (Years)				
<60	18	9	9	0.080
>60	16	3	13	
Location of tumors				
Upper third of esophagus	5	4	1	0.011
Middle third of esophagus	22	4	18	
Lower third of esophagus	7	4	3	
Depth of tumors				
T1/T2	16	8	8	0.151
T3/T4	18	4	14	
Lymph node metastasis				
Without lymph node metastasis	11	7	4	0.026
With lymph node metastasis	23	5	18	
Immunohistochemistry				
PAK1 overexpression in PESCC	34	12	22	0.000
PAK1 overexpression in ANCT	18	16	2	

The correlation between the expression level of PAK1 and different clinical variables was calculated by Fisher’s exact test or χ^2^ test whichever is appropriate. *p* value <0.05 is considered statistically significant; PESCC—Primary esophageal small cell carcinoma; ANCT—Adjacent non-cancerous tissue.

### 2.5. Overexpression of PAK1 Is Associated with Tumor Location and Tumor Metastasis

PAK1 overexpression was significantly associated with tumor location (*p* = 0.011) and lymph node metastasis (*p* = 0.026). Other clinical variables, like age (*p* = 0.080), gender (*p* = 0.271) and depth of tumor (*p* = 0.151), did not show a significant association with PAK1 overexpression. The association of PAK1 with clinical variables is presented in Table 1.

### 2.6. Overexpression of PAK1 Is Associated with Reduced Overall Survival

**Figure 2:**
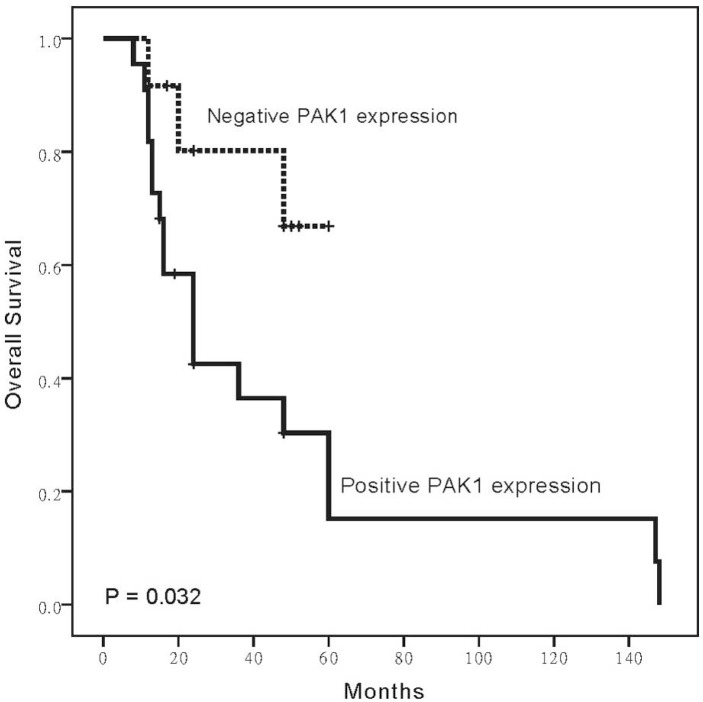
Overexpression of PAK1 influences survival in PESCC patients. PESCC patients showing overexpression of PAK1 had reduced overall survival.

Patients with higher expression level of PAK1 had significantly reduced survival than patients did not show PAK1 expression (*p* = 0.032, Figure 2). The median survival time was 14.5 months (range 2–162 months).

### 2.7. Overexpression of PAK1 Is Associated with γH2AX

**Figure 3:**
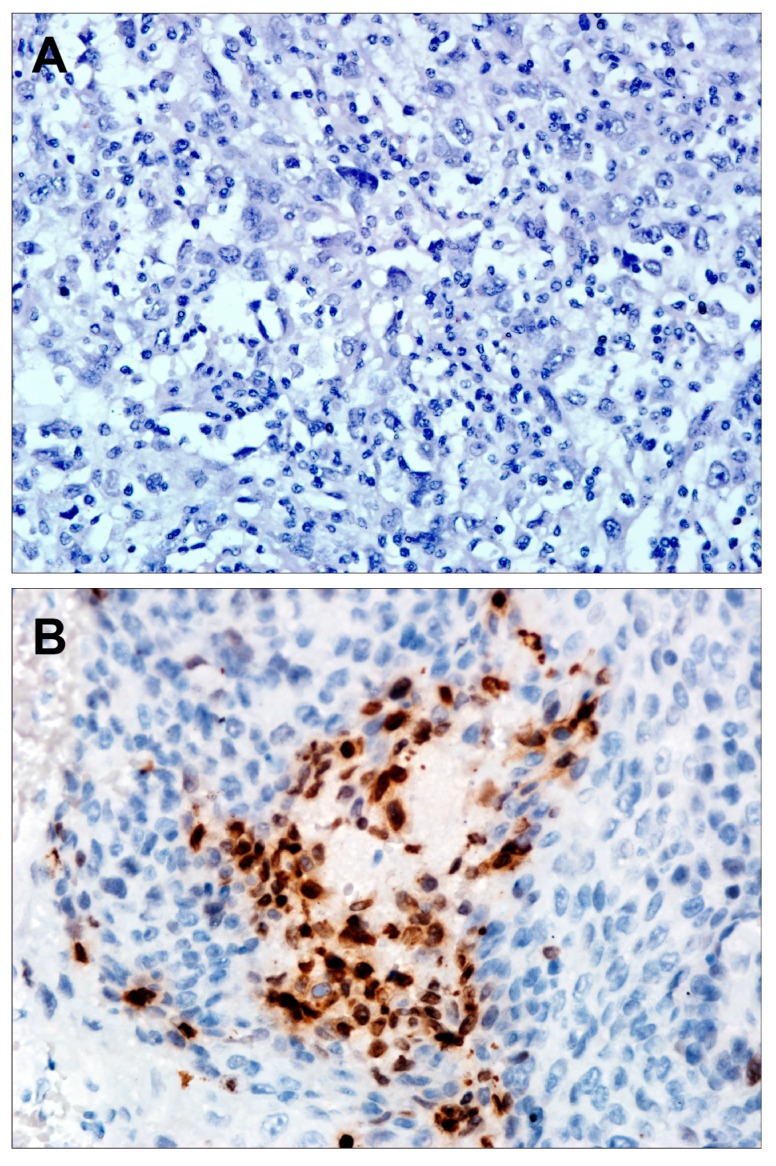
Representative serial sections of PESCC immunohistochemiscal staining for γH2AX: (**A**) negative staining; and (**B**) strong staining. Magnification: 400×.

Because the DNA damage is associated with metastasis [8], and PAK1 regulated γH2AX [27], we thus investigated whether PAK1 is associated with γH2AX in PESCC. We investigated the expression of γH2AX in 24 PESCC patients, and found γH2AX was upregulated in 18 of 24 PESCC (Figure 3).

**Figure 4:**
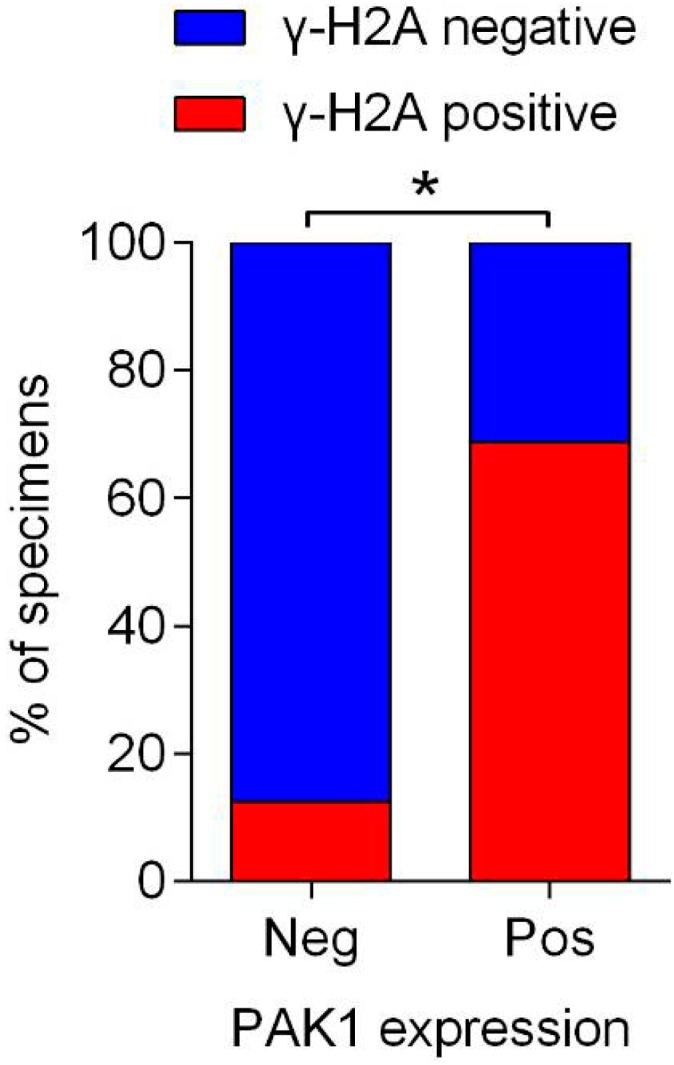
Correlation analysis between PAK1 and γH2AX expression in PESCC. Neg, Negative; Pos, Positive; * *p* < 0.05.

We further examined whether the low or high expression of γH2AX was related to the expression level of PAK1 in 24 PESCC, and found PAK1 expression was positively associated with γH2AX expression, a DNA damage marker (*p* = 0.027) (Figure 4).

### 2.8. Discussion

PESCC is a “rare type” cancer or a rare sub-types of esophageal cancer [31]. Compared with its more common counterpart, PESCC has not yet been well studied. No standard evidence-based approach to treatment has been established, due to the limited knowledge of PESCC [32]. Thus, any efforts to address the relevant issues will be a benefit to our better understanding of the disease and boost the progress of new treatments for these patients.

We here demonstrated the novel evidence that PAK1 was significantly overexpressed in PESCC and the overexpression was significantly associated with tumor location, metastasis and overall survival. In this study, using a relatively large cohort of 34 cases of PESCC, which has been commonly reported with less than 20 cases in English publications, we linked an invasion-regulated kinase PAK1 to DNA damage that is a major cause in cancer progression and metastasis [3,9,10].

PAK1 has potential as a biomarker and target candidate in several aspects. PAK1 is measurable not only in terms of its altered level in tumors *vs.* normal tissues, but is considered to be central to multiple oncogenic networks activated during tumor progression. Protein kinases currently constitute a major focus as potential molecular targets and anti-cancer therapeutics. PAK1, as a protein kinase that contributes to tumorigenesis, is capable of being a drug target. Thus, in addition to being used for diagnosis, prognosis prediction, and therapy surveillance and, more importantly, PAK1 holds promise as a direct target for therapy.

We found that the majority of tumors (64.71%) were located in the middle third of the esophagus, followed by the lower third (20.59%), and finally the upper third (14.70%). Our results are in line with Lu *et al.* [33] who observed high rate of PESCC tumors in the middle third (62.2%), followed by the lower third (27.6%) and the upper third (9.9%) of the esophagus. Similarly, Lv *et al.* [32] observed a higher percent of tumors in the middle third (61.9%), followed by the lower third and the upper third of the esophagus. PESCC arises from endocrine cells, which are unevenly distributed among basal cells of mucosa in epithelial tissues of gastrointestinal tract. In this study, PESCC tumors are predominantly distributed in the middle third, followed by the lower and upper thirds of the esophagus. The underlying reason for this distribution is the abundance of endocrine cells in those regions [34].

In this study, PAK1 was observed to be overexpressed in PESCC tissues (22/34 of cases) in comparison with adjacent non-cancerous tissues (2/18 of cases), confirming its involvement in PESCC. As a condition closely sharing clinicopathological features of extra lung small cell carcinoma, PESCC showed to be histologically indistinguishable from extra lung small cell carcinoma. Ong *et al.* [15] analyzed PAK1 expression in 27 small cell lung cancers and observed that PAK1 was localized only to the cytoplasm and all samples showed weak to moderate PAK1 expression. Similarly, Wang *et al.* [29] observed cytoplasmic overexpression of PAK1 in gastric cancer in comparison with adjacent non-cancerous tissues. In gastric-esophageal junction adenocarcinoma, 72.6% of cases showed cytoplasmic overexpression of PAK1 [19]. Overexpression of PAK1 in cytoplasm of hepatocellular carcinoma in comparison with paired non-cancerous tissues was observed [16]. To add more strength to our observation that PAK1 is involved in tumorigenesis, two functional studies implicated PAK1 in tumorigenesis. Overexpression of kinase active T423E mutant caused anchorage independent growth [35] in breast epithelial cells and mammary oncogenesis and tumor promotion in mouse model [36]. Our finding on PAK1 in PESCC is in agreement with previous studies and, all together, we confirm that PAK1 has a role in tumorigenesis of PESCC.

On the other hand, we found that PAK1 overexpression was also recorded (2/18 of cases) in adjacent non-cancerous tissues. As observed in this study, Li *et al.* [19] also found PAK1 overexpression in adjacent non-cancerous tissues in gastroesophageal junction adenocarcinoma. Likewise, PAK1 overexpression was also reported in 35% of adjacent non-cancerous tissues in gastric cancer by Wu *et al.* [37]. In normal breast epithelial cells, PAK1 expression was absent in comparison with primary breast cancer [15]. Collectively, based on the result that PAK1 was detectable in adjacent non-cancer tissue, it is reasonable to speculate that PAK1 participates in the carcinogenesis of PESCC.

In the present study, of the 23 lymph node metastasis patients, 18 showed PAK1 overexpression. Liu *et al.* [38] observed that, higher level of PAK1 in gastric cancer was associated with metastatic tumor. Likewise, overexpression of PAK1 was associated with lymph node metastasis in gastroesophageal junction cancer [19]. PAK1 was overexpressed in 71% of patients with lymph node metastasis, more than the 43% of patients without lymph node metastasis in gastric cancer [37]. PAK1 protein level was found to be higher in metastatic hepatocellular carcinoma than in corresponding primary hepatocellular carcinoma [16]. Studies observed that overexpression of PAK1 induce cell motility, which in turn causes cells to metastasize to adjacent lymph nodes or organs. Functional studies showed that *in vitro* knock down of endogenous PAK1 by siRNA reduced the motility of cancer cells. In this context, Li *et al.* [37] transfected MKN45 gastric cancer cell line with PAK1 specific siRNA and observed that cells transfected with PAK1 specific siRNA migrated more slowly than cells transfected with control siRNA. Functional studies showed that PAK1 regulated cell motility by phosphorylating LIMK1, which in turn phosphorylates cofilin. Inhibition of cofilin activity inhibits cancer cell motility [39], overexpression of cofilin increases the velocity of cell migration [40]. The available evidence clearly substantiates that the overexpression of PAK1 is positively associated with lymph node metastasis of PESCC tumors.

Kaplan-Meir overall survival analysis of patients with overexpressing PAK1 tumor reveals an association with reduced overall survival. In gastric cancer [29], patients showing PAK1 overexpression had significantly shorter survival than patients that did not show PAK1 expression. In gastroesophageal junction adenocarcinoma [19], shorter survival in patients was associated with overexpression of PAK1. In contrast to these observations, Li *et al.* [19] showed that PAK1 overexpressing patients had better survival than PAK1 lower expressing patients. Underlying reasons that may have contributed to this dissimilarity are cancer types, tumor stage, varied treatment, scoring cells and methodological difference in IHC.

PESCC is thought to originate from multipotential stem cells in the esophageal epithelium, which are the common precursors of esophageal squamous cell carcinoma (ESCC), esophageal adenocarcinoma (EAC) and PESCC [41,42]. Therefore, it sometimes coexists with some ESCC or EAC [41,42,43,44]. It was also described that 8.6% of PESCC coexisted with ESCC in a large cohort of 151 patients [45]. In our patient cohort, only 1 out of 34 (2.94%) cases contained a small amount of ESCC but not EAC, and PAK1 was positively stained in the components of both PESCC and ESCC in this case (Figure S1). According to the preliminary results, PAK1 was also deregulated in the ESCC components and its role in ESCC, a major subtype of esophageal cancer [46,47], remains largely elusive. Therefore, we believe that it is worthy to explore its functions in ESCC in the near future.

Moreover, we investigated the correlation between PAK1 and DNA damage in clinical specimens for the first time, and found that PAK1 expression was positively associated with γH2AX, a DNA damage marker, indicating that PAK1 expression may reflect endogenous genomic instability in tissues. In line with our observations, PAK1 has recently been demonstrated to sufficiently induce the expression of γH2AX [27], and modulate gene expression that was associated with DNA damage induced by ionizing radiation [28]. Moreover, γH2AX has been recently revealed as a prognostic indicator in non-small cell lung cancer [9], endometrial carcinomas [10] and triple-negative breast cancer [11]. Another study has demonstrated the upregulation of γH2AX in metastatic renal cell carcinoma [12].

While there have been many discoveries on DDR, much still remains elusive about the exact mechanism underlying DDR, which is worthy to explore further. Emerging evidence strongly support that excision repair cross-complementing group 1 (ERCC1) plays a critical role in nucleotide excision repair (NER) pathway, and its prognostic and predictive relevance in many cancers, especially in non-small-cell lung cancer have been extensively investigated [48]. Recently, ERCC1 was demonstrated to be an elegant biomarker for adjuvant platinum-based chemotherapy in non-small-cell lung cancer [49], and the expression of ERCC1 has been used to stratify patients with non-small-cell lung cancer and direct the use of platinum-based chemotherapy in clinical trials [50,51]. In the present study, we found PAK1 was a prognostic indicator and potential therapeutic target for PESCC, and our further data indicated a positive association between PAK1 and DNA damage. Combined with the promising prospects of ERCC1, and that small molecular inhibitors targeting PAK1 are currently under evaluation in clinical trials, we truly believe that it is necessary to investigate the relationship between PAK1 and ERCC1 in PESCC in future research. These findings might in the future facilitate the stratification of patients with PESCC based on the evaluation of PAK1 and ERCC1 expression, and direct the use of anti-PAK1 therapies in combination with chemotherapy, which may contribute to the individual treatment of PESCC.

Most publications on PESCC are case reports; little information in literature is linked to the molecular biomarkers or targets [43,52,53]. In this regards, our results clearly showed that PAK1 may be a promising biomarker for PESCC. The growing clinical pipeline of molecular therapy targeting PAK1 using small molecular inhibitors [21,22,23,24,25,26], and also our data suggest the potential usefulness of PAK1 as a molecular therapeutic target in PESCC in future. Although the molecular functions of PAK1 have been described in many aspects [13,14,15,16,17], the regulatory roles of PAK1 on DNA damage/repair have just emerged [27,28]. In this study, we provided evidence of the existence of PAK1-DNA damage pathway in clinical samples for the first time. It is generally conceivable that DNA damage/repair is critical for the carcinogenesis and therapeutic resistance [3]. Up to now, little is known for the molecular mechanism underlying carcinogenesis and progression of PESCC, and due to the lacking of available cell lines, sufficient clinical samples and PESCC related microarray datasets, we have not been able to dissect the underlying mechanisms clearly yet. It has been noticed that PESCC is very sensitive to chemotherapy in the first time treatment and then develops drug resistance afterwards [44]. It is reasonable to suspect that DNA damage/repair may be critical in the progression of PESCC. Thus, further study of PAK1-DNA damage/repair signaling in PESCC in the future is favorable for us to understand PESCC in settings of both molecular mechanism and clinical translation.

## 3. Experimental Section

### 3.1. Patients and Tissue Samples

A total of 34 primary PESCC patients with recorded clinicopathological and follow-up information were recruited from two institutions, both of which are in high-incidence regions with esophageal cancer. Twenty-five cases were diagnosed from November 1997 to September 2012 in the Cancer Hospital of Shantou University Medical College in Chaoshan littoral of Southern China, and nine cases were diagnosed between November 2011 and March 2013 in the Central Hospital of Kaifeng in Henan Province. Institutional human ethics committees of Shantou University Medical College Cancer Hospital and Central Hospital of Kaifeng approved this study. Medical history was recorded for all patients who then underwent physical examinations, such as chest radiograph, barium meal, contrast-enhanced computed tomography scan of the chest and abdomen, complete blood count, blood biochemical parameters, and liver and renal function tests.

We obtained 34 primary PESCC tissues and 18 corresponding adjacent non-cancerous tissues. Tumor and adjacent non-cancerous tissues were fixed in 10% formalin with phosphate buffer (pH 7.4) and subsequently embedded in paraffin. The paraffin embedded tissues were cut into sections of 4 µm and were placed on microscopic glass slides. Thereafter, sections were fixed on glass slides and stained with Hematoxylin and Eosin to establish histological diagnosis and confirmed by immunochemical detection of neuroendocrine markers, including neuron-specific enolase (NSE), synaptophysin (Syn) and chromogranin A (CgA). Tumor samples were histologically confirmed to be PESCC by two pathologists. Tumor staging was performed according to the 7th edition of the American Joint Committee on Cancer (AJCC) staging system for esophageal squamous cell carcinoma [24].

### 3.2. Immunohistochemistry (IHC)

Tissue sections (4 µm) of primary PESCC and adjacent non-cancerous tissues were taken on slides and were initially dewaxed and rehydrated. Antigen retrieval was performed (10 mM Tris/1 mM EDTA, pH 9.0, microwave treated) and then sections were rinsed in Tris buffered saline. Endogenous peroxidase activity was blocked using 100 µL of peroxidase block for 5 min. Sections were incubated with rabbit polyclonal anti-p21 activated kinase 1 antibody (Cell signaling Technology, Boston, MA, USA) or γH2AX (Ser139, Cell Signaling Technology, Boston, MA, USA) for overnight at 4 °C. Tissue sections were washed twice five minutes each in Tris-buffered saline. A volume of 100 µL of 3,3-diaminibenzidine tetrahydrochloride (DAB) was applied on sections for chromogenic detection of PAK1 or γH2AX, then counterstained with Hematoxylin. Sections on slides were sealed by mounting a cover slip with DPX mountant and air dried for 20 min. Positive controls were used in each experiment following supplier’s instructions (Cell signaling Technology). Negative controls applying rabbit IgG to replace primary antibody were also run in each experiment.

### 3.3. IHC Evaluation

Ten random 400× microscopic fields were assessed blind by two independent observers and the average score was taken for analysis. Each specimen was graded with a composite histoscore in a semi-quantitative manner as described by Wang *et al.* [22] and He *et al.* [25]. Staining intensity was categorized as no staining (0), week (1), moderate (2), and strong (3). Likewise, percent of positively stained cells was graded as follows, 0% (0), 1%–25% (1), 26%–50% (2), 51%–75% (3), and 76%–100% (4). To obtain a composite histoscore for each specimen, the average values for stain intensity was multiplied by average values for percent of positively stained cells (*i.e.*, histoscore = percent of positively stained cells × stain intensity). Based on this histoscore point, specimens with 3+ histoscore considered positive while specimens with ≤3 considered negative.

### 3.4. Statistical Analysis

Multivariate analysis was performed by Cox proportional hazard regression model to investigate prognostic importance of the following clinical variables such as gender, age (<60 years *vs.* >60 years), tumor location (Upper third/Middle third/lower third of esophagus), depth of tumor (T1/T2; T3/T4) and regional lymph node metastasis (N0/N1 to N3). The difference in the expression level of PAK1 was correlated with different clinical variables and γH2AX through Fisher’s exact test or χ^2^ test, whichever is appropriate. Univariate analysis to estimate survival probability in patient subgroups was carried out by Kaplan-Meier method and log-rank test was performed to statistically compare the survival curves of patient subgroups. Overall, survival time was calculated from the date of surgery and date of patient death or last follow-up. All statistical tests were performed two-sided and a *p* value <0.05 considered statistically significant. All the statistical analyses were performed by using SPSS software V13.0 (SPSS Inc., Chicago, IL, USA).

## 4. Conclusions

We show for the first time that PAK1 was frequently overexpressed in PESCC and in addition, overexpression of PAK1 was significantly associated with tumor location, metastasis and reduced overall survival, and γH2AX, a DNA damage marker. Hence, potential of PAK1 as a potential target for prognosis and molecularly targeted therapy in PESCC is worth considering in future studies looking for intervention of this dreadful carcinoma.

## Supplementary Materials

Supplementary materials can be found at http://www.mdpi.com/1422-0067/16/06/12035/s1.
