# Supplementary material for: Dysregulation of PAK1 Is Associated with DNA Damage and Is of Prognostic Importance in Primary Esophageal Small Cell Carcinoma
Source: Int J Mol Sci. 2015 May 27;16(6):12035–50. doi: 10.3390/ijms160612035 (PMC4490427; doi:10.3390/ijms160612035)
Supplement: Supplementary file 1 [file ijms-16-12035-s001.pdf]

Supplementary Information

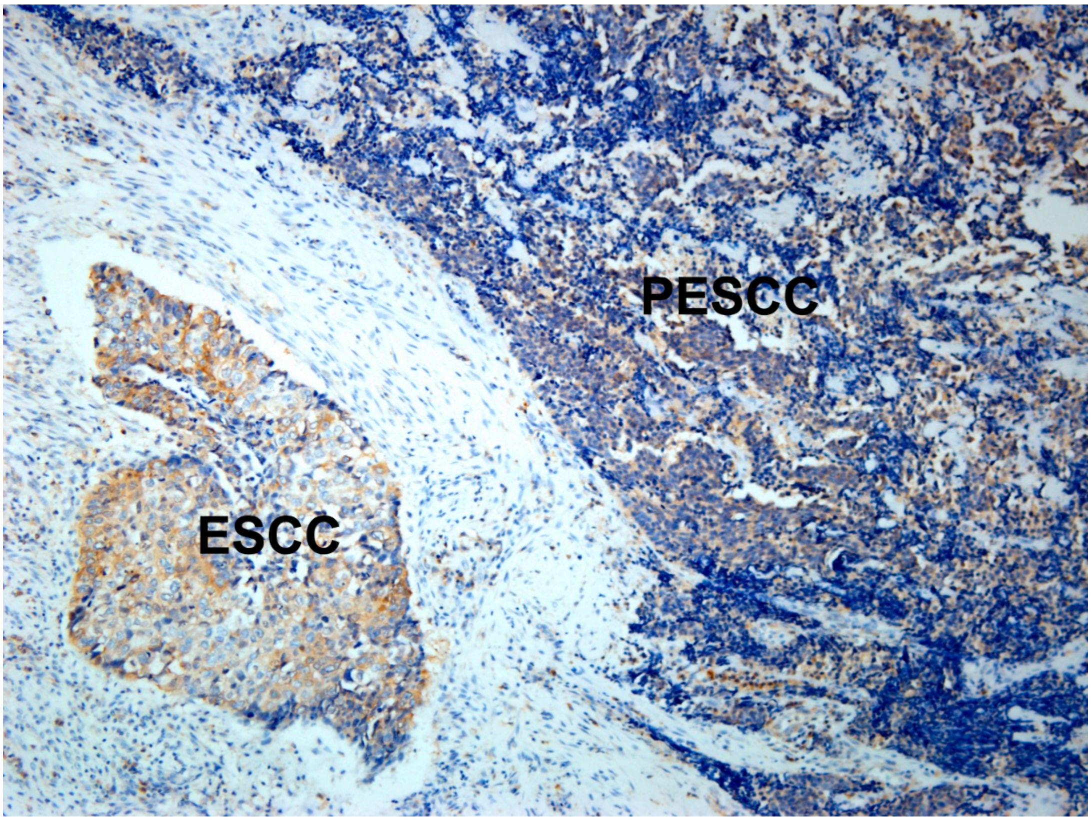

Figure S1. Representative image of immunohistochemiscal staining for PAK1 in the components of both PESCC and ESCC. Magnification (×100)
